# High-level HIV-1 Nef transient expression in *Nicotiana benthamiana *using the P19 gene silencing suppressor protein of Artichoke Mottled Crinckle Virus

**DOI:** 10.1186/1472-6750-9-96

**Published:** 2009-11-20

**Authors:** Raffaele Lombardi, Patrizia Circelli, Maria Elena Villani, Giampaolo Buriani, Luca Nardi, Valentina Coppola, Linda Bianco, Eugenio Benvenuto, Marcello Donini, Carla Marusic

**Affiliations:** 1 Centro Ricerche Casaccia, Via Anguillarese 301, I-00123, Rome, Italy; 2 Centro Ricerche Trisaia, SS 106 Ionica, I-75026, Rotondella (Matera), Italy

## Abstract

**Background:**

In recent years, different HIV antigens have been successfully expressed in plants by either stable transformation or transient expression systems. Among HIV proteins, Nef is considered a promising target for the formulation of a multi-component vaccine due to its implication in the first steps of viral infection. Attempts to express Nef as a single protein product (not fused to a stabilizing protein) in transgenic plants resulted in disappointingly low yields (about 0.5% of total soluble protein). In this work we describe a transient expression system based on co-agroinfiltration of plant virus gene silencing suppressor proteins in *Nicotiana benthamiana*, followed by a two-step affinity purification protocol of plant-derived Nef.

**Results:**

The effect of three gene silencing viral suppressor proteins (P25 of Potato Virus X, P19 of either Artichoke Mottled Crinckle virus and Tomato Bushy Stunt virus) on Nef transient expression yield was evaluated. The P19 protein of Artichoke Mottled Crinckle virus (AMCV-P19) gave the highest expression yield in vacuum co-agroinfiltration experiments reaching 1.3% of total soluble protein, a level almost three times higher than that previously reported in stable transgenic plants. The high yield observed in the co-agroinfiltrated plants was correlated to a remarkable decrease of Nef-specific small interfering RNAs (siRNAs) indicating an effective modulation of RNA silencing mechanisms by AMCV-P19. Interestingly, we also showed that expression levels in top leaves of vacuum co-agroinfiltrated plants were noticeably reduced compared to bottom leaves. Moreover, purification of Nef from agroinfiltrated tissue was achieved by a two-step immobilized metal ion affinity chromatography protocol with yields of 250 ng/g of fresh tissue.

**Conclusion:**

We demonstrated that expression level of HIV-1 Nef in plant can be improved using a transient expression system enhanced by the AMCV-P19 gene silencing suppressor protein. Moreover, plant-derived Nef was purified, with enhanced yield, exploiting a two-step purification protocol. These results represent a first step towards the development of a plant-derived HIV vaccine.

## Background

Plants are an advantageous system for the cost-effective expression of large amounts of safe complex recombinant proteins. In particular, it has been extensively demonstrated that plants are ideal bioreactors to classical heterologous protein expression systems for the production of functional monoclonal antibodies, enzymes and vaccine components [1]. The major advantages of plants over traditional expression systems based on bacterial and mammalian cells are a reduced risk of contamination by human pathogens and low costs, especially for large-scale production [2]. In recent years, it has been demonstrated that both structural and regulatory proteins of HIV or related viruses can be successfully expressed in plants by either stable transformation (nuclear or plastid transformation) or transient expression systems (plant virus vectors and leaf agroinfiltration) [3]. Different structural HIV proteins have been successfully expressed in transgenic plants including Pr55Gag, Gag p24 and p24/p17 [4-7]. The non-structural HIV-1 accessory protein Nef is considered a good candidate for the formulation of vaccines that combine both structural and functional viral components. In fact, during the viral life cycle, Nef is expressed early and is essential to both viral load and disease progression. Moreover, *nef *genes are highly conserved in all primate lentiviruses (HIV-1, HIV-2, SIV) and data show that patients classified as long-term non-progressors bear alterations in the *nef *gene [8]. Encouraging studies on the production of HIV vaccines containing Nef have been recently published. A clinical evaluation of a multi-component vaccine containing recombinant gp120 and Nef-Tat fusion proteins was performed in uninfected human volunteers [9]. Moreover, the effects of a genetic vaccine combining both structural (Gag/Pol, Env) and regulatory (Rev, Tat, Nef) viral proteins were evaluated in the SIV-*Macaca *animal model [10,11].

Nef has been already produced in transgenic plants (nuclear or plastid transformation) using different expression strategies and different signal or fusion peptides to direct the expression of the viral protein either to the cytosol or secretory pathway. The most successful strategies were those in which Nef was fused to stabilizing proteins as the chimeric protein zeolin or the HIV p24 antigen [7,12,13]. In particular, Nef fused to p24 yielded the highest levels of recombinant protein in transplantomic plants (about 40% of total soluble protein (TSP)) [14]. On the other hand, the expression of Nef alone in plant cells proved to be very difficult and the most promising results were obtained expressing a mutated form of Nef in which the N-terminal myristoyl-acceptor glycine (Gly2) was mutated to alanine (Nef p27 mut). This non-myristoylated derivative accumulated up to 0.5% TSP in the cytosol of transgenic *Nicotiana tabacum *(*N. tabacum*) [15]. Indeed, it was previously shown that recombinant Nef lacking myristoylation was able to elicit an enhanced cellular immunity compared to its wild-type counterpart, representing an ideal candidate for the formulation of multicomponent vaccines [16].

In order to investigate novel trategies to enhance Nef p27 mut yield, a transient expression system based on leaf agroinfiltration of *Nicotiana benthamiana (N. benthamiana) *plants has been devised. Transient expression systems rely either on the epichromosomal expression of *Agrobacterium tumefaciens *(*A. tumefaciens*) directly infiltrated into plant tissues (agroinfiltration) or on viral-based expression vectors. Both systems generally offer several advantages over the generation of transgenic plants, including production speed and high expression yields. Among transient expression strategies, agroinfiltration system has been used to accumulate high levels of different heterologous proteins, ranging from reporter genes (green fluorescent protein and β-glucuronidase) [17-19] to complex multimeric molecules, such as immunoglobulins [19-23]. Main advantages of this system consist in the possibility of expressing long gene sequences, flexibility and ease of expressing more than one gene simultaneously in the same cell, allowing efficient assembly of multimeric proteins. Moreover, this system could be easily applied to industrial scale for massive production of recombinant proteins. A major drawback of this technology is that expression levels of heterologous proteins are generally limited by the post-transcriptional gene silencing (PTGS) response that may take place in the infiltrated plant tissue [24]. To overcome this limitation, the use of viral suppressors of gene silencing in the agroinfiltration assays was able to prevent PTGS and enhance transient expression levels of heterologous proteins. This was clearly confirmed in *N. benthamiana *using the P19 suppressor of gene silencing of Tomato Bushy Stunt virus (TBSV-P19), which was able to enhance expression yields of a range of proteins up to 50 folds [17].

In this work we describe the transient expression of Nef p27 mut [15] (that will be indicated as Nef throughout the text) in *N. benthamiana*, using a vacuum-agroinfiltration system. To enhance expression levels, the co-agroinfiltration of Nef in combination with three different viral suppressors of gene silencing (P25 of Potato Virus X -PVX-P25-, TBSV-P19 and AMCV-P19) was evaluated. Results demonstrated that AMCV-P19 gave the highest Nef yields with an almost three-fold increase compared to those previously reported in stable transgenic plants. The effect of these three different viral proteins on Nef accumulation in leaves at different positions on the plant (top, middle, bottom) was also assessed. Moreover, an optimised Nef purification protocol, based on a two-step immobilized-metal affinity chromatography (IMAC) was also performed.

## Results

### Cloning of the AMCV-P19 gene silencing suppressor

**Figure 1:**
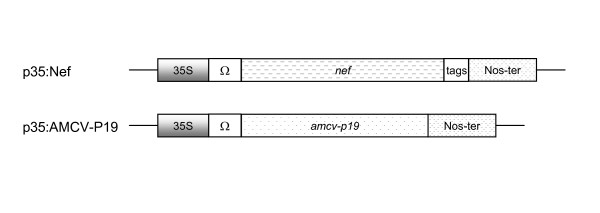
**Schematic representation of the p35:Nef and p35:AMCV-P19 constructs**. In p35:Nef and p35:AMCV-P19 constructs, *nef *and *amcv-p19 *genes are under the control of the CaMV 35S promoter (35S) and the TMV 5'-untranslated leader sequence omega (Ω). *nef*: cDNA encoding a mutated form of Nef in which the myristoylation consensus sequence is abolished; *amcv-p19*: cDNA encoding AMCV-P19 gene silencing suppressor; tags: Flag-tag followed by a 6× histidine tag.

AMCV viral cDNA was retrotranscribed from genomic RNA extracted from purified virus particles as previously described [25]. The *amcv*-*p19 *gene was amplified from viral cDNA by polymerase chain reaction (PCR), using specific primers flanking the *p19 *coding region [26]. The unique amplified product was cloned into the pBI-Ω plant expression vector under the control of Cauliflower mosaic virus (CaMV) 35S promoter and the nopaline synthase (NOS) terminator sequence [15]. To enhance translational efficiency, the Tobacco Mosaic Virus (TMV) 5'-untranslated leader sequence (omega, Ω) was fused in frame with the heterologous gene (*amcv-p19*). The p35:AMCV-P19 and p35:Nef constructs (Figure 1) were electroporated into *A. tumefaciens *strain LBA4404 and used in co-agroinfiltration experiments.

### Nef transient expression

**Figure 2:**
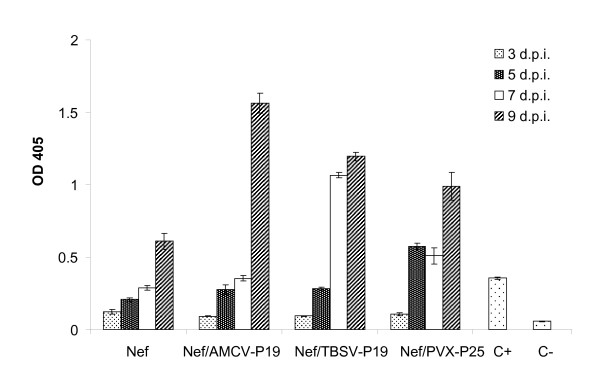
**Time course expression analysis of Nef in vacuum agroinfiltrated *N. benthamiana *plants**. Six weeks old *Nicotiana benthamiana *plants were vacuum-infiltrated with *Agrobacterium. tumefaciens *clones harbouring Nef, Nef/AMCV-P19, Nef/TBSV-P19 and Nef/PVX-P25. Leaf samples from three different plants were collected on 3, 5, 7, and 9 days post infiltration (d.p.i.). Leaf extracts were normalised for total soluble protein content (TSP) and analysed by direct ELISA using a mouse monoclonal antibody to HIV-1 Nef. For all samples a total of 50 μg of TSP were loaded in each ELISA plate well. C+: 100 ng of bacterially-derived Nef; C-: leaf extract of plants infiltrated with infiltration buffer. Values are the mean ± standard error of the mean (SEM) of triplicate samples.

**Figure 3:**
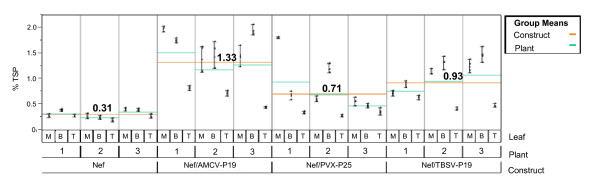
**Nef expression analysis represented as variability chart for three-factors (Construct-Plant-Leaf position crossed data)**. The effect of different viral suppressors of gene silencing and influence of leaf position on Nef expression yield was evaluated by ELISA using a mouse monoclonal antibody against HIV-1 Nef. For this study three independent plants were agroinfiltrated with Nef, Nef/AMCV-P19, Nef/PVX-P25 or Nef/TBSV-P19 and leaf samples were collected at day 9 post infiltration from different positions on the plant (T: top leaf; M: middle leaf; B: bottom leaf). Leaf extracts (50 and 25 μg of total soluble protein (TSP)) were loaded on the ELISA plate wells as four indipendent replicas. All data were subjected to analysis of variance (ANOVA) using JMP8 Statistical Discovery software. The variability chart reports the maximum and minimum bars to show the range of measurements. Nef yield is expressed as percentage of total soluble protein (% TSP). The group means are represented by orange (Construct) and green (Plant) lines as reported in the legend. Mean values of Nef yield (% TSP) for each construct group are reported on the chart over the orange line.

Six weeks old *N. benthamiana *plants were infiltrated with the *A. tumefaciens *clone harbouring Nef alone or with mixed cultures of *Agrobacterium *carrying p35:Nef and p35:AMCV-P19 (Nef/AMCV-P19), p35:TBSV-P19 (Nef/TBSV-P19) or p35:PVX-P25 (Nef/PVX-P25) expression cassettes. In order to determine the peak of Nef expression in the agroinfiltrated *N. benthamiana *plants, leaf samples were collected from three independent plants on 3, 5, 7, and 9 days post infiltration (d.p.i.) (Figure 2). Nef expression levels were assayed by direct ELISA and the results of these experiments showed that the highest expression levels using gene silencing suppressors were reached at 9 d.p.i. in all samples (Figure 2). Based on this preliminary results we set up a vacuum-agroinfiltration transient expression experiment in order to evaluate and compare the effect of the different gene silencing suppressors as well as the influence of leaf age/position on Nef yield. Three plants per construct were agroinfiltrated at the six-leaf stage and at 9 d.p.i. leaves at three different positions, corresponding to the first node counting from the top (top leaf, T), the third (middle leaf, M), and the fifth node (bottom leaf, B), were collected. Plant extracts have been analysed by ELISA and data obtained were subjected to analysis of variance (ANOVA). As expected, co-agroinfiltration with the three viral suppressors of gene silencing enhanced Nef expression levels (Figure 3). Moreover, the comparison among gene silencing suppressors, revealed that AMCV-P19 produced the highest Nef yield with a mean value of 1.33% TSP (Figure 3) representing a 4.4 fold increase compared to Nef alone. The differences observed in Nef expression levels using AMCV-P19, TBSV-P19 or PVX-P25 resulted statistically significant (P < 0.0001). *N. benthamiana *plants co-agroinfiltrated with Nef/AMCV-P19 showed 1.4 and 1.8 fold increase in Nef yield compared to plants co-agroinfiltrated with Nef/TBSV-P19 and Nef/PVX-P25, respectively. Most interestingly, expression yield analysis performed on leaves at different positions, demonstrated a statistically significant decrease (P < 0.0001) of Nef expression levels in top (young) leaves compared to middle/bottom leaves in all constructs (Figure 3). Furthermore, no statistically significant (P = 0.54) difference in Nef expression yield was evidenced between the three replica plants used in this study for all constructs (Figure 3).

### Suppression of local RNA silencing by AMCV-P19

**Figure 4:**
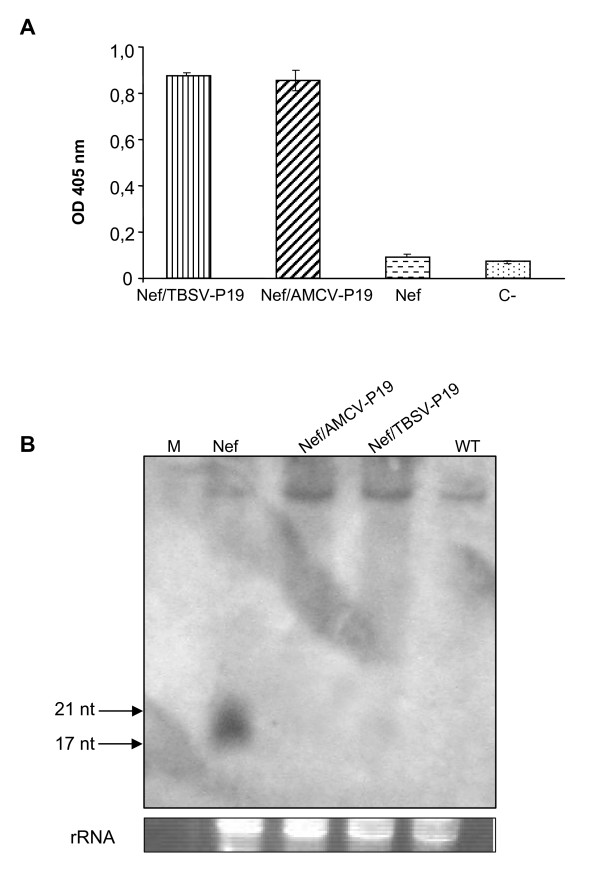
**Correlation between P19 protein accumulation and levels of Nef specific siRNAs**. (A) ELISA of leaf extracts from *Nicotiana benthamiana *plants agroinfiltrated with Nef, Nef/TBSV-P19 and Nef/AMCV-P19 was performed using a rabbit polyclonal antibody against TBSV-P19. Leaves were collected at 9 d.p.i. and plant extracts were normalized for total soluble protein (TSP). Fifty micrograms of TSP were loaded in each ELISA plate well. Values are means of triplicate determinations ± standard errors of the means. C-: leaf extract of plants infiltrated with infiltration buffer. (B) Detection of Nef specific siRNAs. Total RNA (15 μg) extracted from leaves agroinfiltrated with Nef, Nef/AMCV-P19, Nef/TBSV-P19, and from mock infiltrated plant used as a control (WT), was separated on denaturing 15% (w/v) polyacrylamide gel with 8 M Urea, stained with ethidium bromide to display relative amounts of rRNA and transferred to a positively charged nylon membrane. Nef specific digoxigenin-labelled RNA (+) (618 nt) was used as probe. M: si RNA low molecular weight RNA marker (synthetic siRNA duplexes 17, 21 and 25 bp).

To verify the correlation between AMCV-P19 expression and the accumulation levels of *nef *specific siRNAs, *N. benthamiana *leaves were either infiltrated with an *Agrobacterium *suspension carrying *nef *gene alone or with mixed cultures of *Agrobacterium *carrying p35:Nef (Nef) and p35:AMCV-P19 (Nef/AMCV-P19) or p35:TBSV-P19 (Nef/TBSVP-19) expression cassettes. Leaf samples were collected at 9 d.p.i. and analysed by ELISA and Northern blot. The expression of AMCV or TBSV-P19 gene silencing suppressors in the plant extracts was assayed by ELISA, using a polyclonal antibody to TBSV-P19 (Figure 4A) demonstrating an accumulation in agroinfiltrated leaves. Moreover, total RNA from the same samples was extracted and separated on denaturing Polyacrylamide Gel Electrophoresis (PAGE) and analysed by Northern blot using a sense RNA probe (618 nt) corresponding to the *nef *gene. As shown in figure 4B, *nef *specific siRNAs (of about 17-21 nt) accumulated only in the leaves infiltrated with *Agrobacterium *carrying *nef *gene alone while siRNAs were not detected in the Nef/AMCV-P19 or Nef/TBSV-P19 co-agroinfiltrated leaves (Figure 4B). These results demonstrated that P19 expression in the agroinfiltrated tissue significantly reduced the accumulation of silencing-associated *nef *specific siRNAs.

### Two-step purification of plant-derived Nef

**Figure 5:**
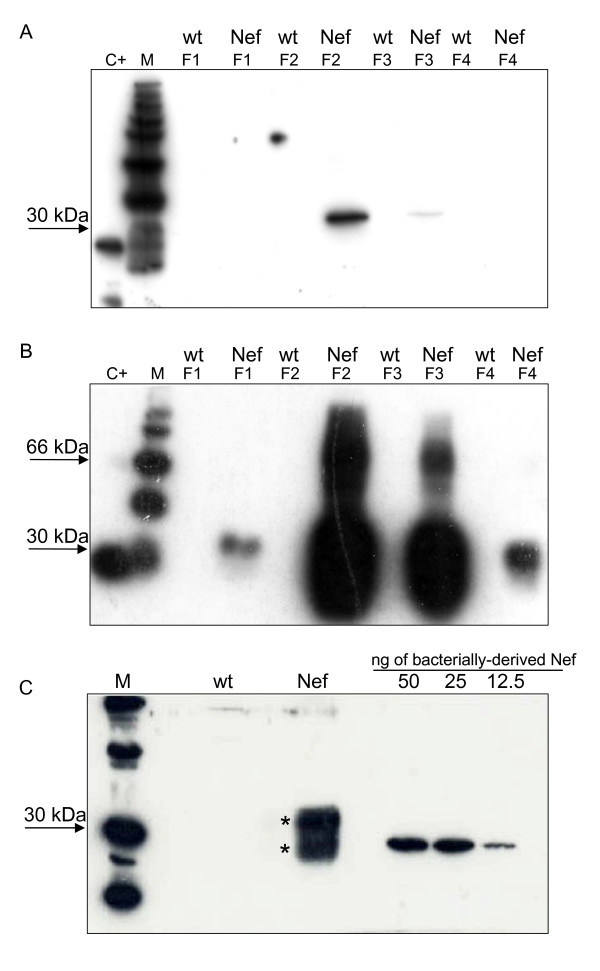
**Purification of plant-derived Nef**. Western-blot analysis of purified Nef extracted from: (A) transgenic *Nicotiana tabacum *leaves or (B) *Nicotiana benthamiana *agroinfiltrated leaves with Nef/AMCV-P19. Total leaf protein homogenates were purified using a two step immobilized-metal affinity chromatography (IMAC) protocol consisting of a nickel-nitrilotriacetic acid (Ni-NTA) column and a cobalt column. Aliquots (20 μl) from four elution fractions (F1-F4) were loaded on reducing SDS-12% (w/v) polyacrylamide gel (PAGE) and analysed by Western-blot using a monoclonal antibody (mAb) against Nef. F1-F4 Nef: elution fractions of Nef transgenic tobacco (A) or agroinfiltrated *N. benthamiana *plants (B). C+: 20 ng of *E. coli*-derived Nef. Bands at about 30 kDa correspond to the monomeric form of Nef, while the bands at 66 kDa indicate the presence of Nef dimers. (C) Western-bolt analysis of Nef purified from agroinfiltrated *N. benthamiana *leaves (10 g of fresh weight). Fractions F2, F3 and F4 (0.5 ml each) were pooled and a 20 μl aliquot was loaded on reducing SDS-12% (w/v) polyacrylamide gel (PAGE). Two distinct bands at about 30 kDa are indicated by asterisks. The upper band corresponds to monomeric Nef, while the lower band proved to be a degradation product as revealed by mass spectrometry. Plant-derived Nef yield (average of 250 ng per gram of fresh leaf weight) was estimated by densitometric analysis of the upper band obtained in Western-blot.

In order to optimize the purification protocol of plant expressed Nef fused to a hexa-histidine tag (His 6-tag) [15], a two step IMAC experimental procedure was set up using both a nickel-nitrilotriacetic acid (Ni-NTA) and cobalt affinity purification columns. Plant extracts were prepared from 10 g of both transgenic *N. tabacum *plant leaves (line 64) expressing Nef [15] and *N. benthamiana *fresh leaves agroinfiltrated with Nef/AMCV-P19. Nef purification was performed in parallel from both extracts using the same protocol. As negative controls, untransformed (wt) *N. tabacum *and non-infiltrated (wt)*N. benthamiana *plants were used. The homogenates were clarified by centrifugation, supernatants were loaded onto a Ni-NTA column and the eluted fractions containing Nef were then passed through a desalting column for buffer exchange. Samples derived from the first purification step were further separated through a cobalt affinity purification column and the eluted fractions were analysed by Western blot (Figure 5A and Figure 5B). The results clearly showed a strong increase of purified Nef yield (Nef monomer corresponding to the band at about 30 kDa) in all four elution fractions from agroinfiltrated samples (Figure 5B) compared to those from transgenic leaves (Figure 5A).

In two elution fractions (F2 and F3) obtained after purification from agroinfiltrated leaves, high molecular weight bands were also observed (~66 kDa) (Figure 5B) probably due to the ability of Nef core to form dimers and trimers, as previously reported [27]. On the contrary, the formation of Nef multimers was not observed in transgenic plants probably due to the extremely low expression yields reported in this system. Purified plant-derived Nef has an additional flag-histidine tag fused at its C-terminus and, therefore, showed a higher molecular weight compared to the bacterially-derived Nef used as a positive control [15]. Purified Nef from agroinfiltrated *N. benthamiana *leaves revealed the presence of two distinct bands indicated by asterisks in figure 5C. The upper band corresponded to intact plant-purified Nef, while the lower band proved to be a degradation product, as indicated by mass spectrometry (MS) (data not shown). The amount of purified Nef from both transgenic tobacco and agroinfiltrated *N. benthamiana leaves *was estimated by densitometric analysis of the bands obtained in Western-blot using the image J v. 1.36b software. A standard curve was obtained from the band densities of three different dilutions (12.5, 25 and 50 ng) of bacterially-derived Nef (Figure 5C). Calculated average purification yields were of 30 and 250 ng per gram of fresh transgenic and agroinfiltrated leaves, respectively.

## Discussion and conclusion

We have previously shown that HIV-1 Nef protein lacking the myristoylation signal can be successfully expressed in the cytosol of transgenic tobacco plants accumulating up to 0.5% TSP [15]. Data on Nef plant expression using different approaches, all based on stable transformation, have been reported [3] showing that the highest yields (40% TSP) were generally obtained by fusing Nef to stabilizing protein components [12-14]. However, efficient extraction and purification of the antigen expressed as a fusion protein, still represents a major issue.

In our work we focused our efforts to enhance the production of Nef (as a single protein) in the cytoplasm of plant cells. To our knowledge, no attempt of transiently expressing Nef as a single gene has been reported in literature until now. Therefore, with the aim of increasing the expression levels of plant-produced Nef, we assayed an efficient vacuum-agroinfiltration system in *N. benthamiana *boosted by different viral suppressors of gene silencing (AMCV-P19, TBSV-P19, PVX-P25). Previous works already showed that the use of P19 derived from TBSV enhanced expression levels of different proteins in *N. benthamiana *up to 50 fold [17]. In this work we performed a quantitative detailed analysis to evaluate and compare the effect of different gene silencing suppressors as well as the influence of leaf position on yield of the foreign protein. The results illustrated herein, demonstrated that leaves co-agroinfiltrated with Nef and AMCV-P19 gave the highest yield (mean value of 1.3% TSP). This represented a 4.4 fold increase of Nef expression levels compared to plants infiltrated without AMCV-P19, while a 3 and 2.2 fold increase was observed in the case of TBSV-P19 and PVX-P25, respectively. The significant difference in expression yield between AMCV-P19 and TBSV-P19 is somehow unexpected considering their high aminoacid sequence homology (about 90%). Moreover, we observed that Nef expression level using both AMCV and TBSV-P19 proteins was substantially higher compared to that obtained with PVX-P25, in accordance to previously reported data [17]. Furthermore, no significant synergic effects in enhancing the expression levels were detected by co-infiltrating plants with a combination of recombinant *Agrobacterium *strains carrying either AMCV-P19/PVX-P25 or TBSV-P19/PVX-P25 (data not shown). This could be explained in light of what observed in siRNA detection experiments in which both P19 proteins totally hampered the accumulation of Nef specific siRNAs.

Expression analysis performed on leaves at different positions, demonstrated a statistically significant decrease of Nef expression levels in top leaves compared to middle/bottom leaves for all costructs. These data are in contrast with what has been observed in transgenic plants expressing antibodies in which top leaves generally showed the highest expression levels [28,29]. This behaviour is probably due to the higher protein degradation rate occurring in mature leaves. It is conceivable that degradation processes may have scarce influence in transient expression systems, as the time frame of recombinant proteins expression is limited to just 9 days before harvesting. Therefore, the lower expression level observed in top leaves is an important issue to consider when using transient agroinfiltration systems for recombinant protein production.

In this work we demonstrated that protein yield obtained by transient expression of Nef alone in *N. benthamiana *was lower (0.31% TSP) compared to what previously reported in *N. tabacum *transgenic plants (0.5% TSP) [15]. A direct comparison of Nef accumulation levels in agroinfiltrated leaves using a viral suppressor of gene silencing and transgenic plants cannot be made, nevertheless, we showed that transient expression using AMCV-P19 is an advantageous system for rapid and high yield production of Nef. Furthermore, a two-step IMAC protocol was used to optimize Nef purification from both agroinfiltrated and transgenic plant leaves. An eight-fold increase of purification yield from agroinfiltrated *N. benthamiana *(250 ng/g fresh tissue) with respect to transgenic tobacco (30 ng/g fresh tissue) was observed. This striking difference was somehow unexpected considering that the calculated Nef expression yield in agroinfiltrated plants resulted only about three times higher than in transgenics. A possible explanation could be that different plant species (*N. tabacum *or *N. benthamiana*) were utilised for purification, and in the case of *N. benthamiana *extracts, Nef showed a diminished tendency to aggregate and precipitate in the insoluble protein fraction. In fact, during extraction and purification a consistent part of the protein (about 30-40%) was lost due to aggregation and precipitation phenomena.

Taken together, the results presented here suggest that the use of viral suppressor of gene silencing, AMCV-P19, could efficiently enhance the transient expression of recombinant proteins in *N. benthamiana*. Moreover, this transient expression system provides the advantage of higher production speed over the generation of transgenic plants.

## Methods

### Gene engineering

All genes were cloned under the control of the constitutive CaMV 35S promoter and the NOS terminator sequence. The cloning strategy of the p35:Nef construct (Figure 1) carrying *nef-p27mut *gene was previously described [15]. For generation of the p35:AMCV-P19 constructs, the *amcv*-*p19 *coding sequence was amplified from the viral genome (GenBank Accession NC_001339). Briefly, viral RNA was extracted from AMCV purified virus particles as described before [25], and was used as the template for oligo(dT)-primed first-strand synthesis of cDNA. The *Bam*HI-Forward (5'-CGGGATCCATGGAGCGAGTTATACAAGG-3') and *Sma*I-Reverse (5'-TCCCCCCGGGCTACTCGCTTTCTTCTTTGAAG-3') primers (restriction sites underlined) were used to amplify *the p19 *gene by PCR using Pfu polymerase (Stratagene, La Jolla, CA). The resulting PCR product was cloned into the pBI-Ω plant expression vector [15] generating the p35:AMCV-P19 construct (Figure 1). Constructs p35: TBSV-P19 and p35: PVX-P25 carrying the *tbsv*-*p19 *and *pvx*-*p25 *genes respectively [17], were kindly provided by Prof David Baulcombe (University of Cambridge). All constructs were electroporated into *A. tumefaciens *strain LBA4404.

### Transient expression in *N. benthamiana*

Transient expression in *N. benthamiana *was performed by vacuum agroinfiltration. *A. tumefaciens *clones harbouring the constructs p35:Nef, p35:AMCV-P19, p35:TBSV-P19 and p35: PVX-P25 were grown separately. Bacteria were pelleted by centrifugation at 4000 g, resuspended in infiltration buffer [10 mM 2-(N-morpholino) ethanesulphonic acid (MES), 10 mM MgSO4, pH 5.8] to a final OD_600 _value of 0.6. The *A. tumefaciens *cultures used in the co-agroinfiltrations of Nef/AMCV-p19, Nef/TBSV-p19 and Nef/P25 were prepared by mixing different bacterial cultures bearing each type of plasmid but keeping a final OD_600 _of 0.6 for each one. Six weeks old *N. benthamiana *plants (at the 6 leaf stage) were infiltrated by completely submerging each plant in the *Agrobacterium*-cultures inside a desiccator (Secador Technidome, Sigma-Aldrich, USA) or just in infiltration buffer (mock, negative control). Vacuum was then applied using a vacuum pump (Savant VP190) reaching ~10 mm Hg and then quickly released. Infiltration was confirmed visually, observing infiltrated areas as translucent. Plants were then placed in the greenhouse and leaf sampling was performed at 3, 5, 7, 9 days post infiltration (d.p.i.) and the material was frozen in liquid nitrogen and stored at -80°C. For quantitative ELISA, sampling of the leaves was performed only at 9 d.p.i. and leaves at three different positions on the plant, corresponding to the first node counting from the top (top leaf, T), the third (middle leaf, M), and the fifth node (bottom leaf, B), were collected. All plants were grown at 25°C under 16 h light/8-h dark photoperiod.

### ELISA detection of plant-derived Nef and P19

Nef and P19 expression was evaluated by direct ELISA. The total soluble protein (TSP) were obtained as described: for each sample 100 mg of frozen leaf tissues were homogenized with an Ultra-Turrax homogenizer T25 (IKA, Staufen, Germany) in 250 μl of ice-cold PBS-buffer (PBS) containing protease inhibitors (Complete™ EDTA Free, Roche, Germany). Extracts were clarified by centrifugation at 20000 g for 30 min at 4°C. The amount of TSP was estimated using the Bradford colorimetric assay as specified by the manufacturer (Bio-Rad, Hercules, CA, USA). ELISA assays were performed coating 96-well microplates with 50 μg of total protein extract for each sample in three replicas. Bacterially-derived Nef (EVA#650, NIBSC-CFAR MRC) was used as positive control. The plates were blocked with 5% (w/v) dry milk in PBS. After washing with 0.1% (v/v) Tween 20 in PBS, Nef was detected using a mouse monoclonal antibody (mAb) to HIV-1 Nef (EVA#3067.4, NIH AIDS Research and Reference Reagent Program) diluted 1:500 in 2% (w/v) dry milk in PBS. The ELISA plate was incubated for 2 h at 37°C. After a second wash with 0.1% (v/v) Tween 20 in PBS, a secondary horseradish peroxidase (HRP) conjugated anti-mouse antibody (NXA931 GE Healthcare, UK) at 1:2500 dilution in 2% (w/v) dry milk in PBS was used. The ELISA plate was incubated for 1 h at 37°C. P19 was detected using a rabbit polyclonal antibody to TBSV-P19 diluted 1:1000 in 2% (w/v) dry milk in PBS. ELISA plate was incubated for 2 h at 37°C. After washing with 0.1% (v/v) Tween 20 in PBS, a secondary Biotin-Labeled Affinity purified anti-rabbit antibody (Cat# 16-15-06 KPL, USA) at 1:2500 dilution together with streptavidin - HRP conjugate (RPN 1231 Amersham Biosciences, UK) at 1:2000 dilution in 2% (w/v) dry milk in PBS, were used. The ELISA plate was incubated for 1 h at 37°C. Enzymatic activity was measured using 2,2-azino-di-3-ethylbenz-thiazoline sulphonate substrate (ABTS KPL, USA). The reactions were stopped after 30 min at room temperature by adding ABTS stop solution (KPL, USA) and the plate was read at 405 nm using a microtitre plate reader (TECAN-Sunrise, Groedig, Austria)

Quantitative ELISA of agroinfiltrated plant extracts with Nef only or in combination with the three different gene silencing suppressors, was performed on leaf samples collected at 9 d.p.i. Three plants per construct were agroinfiltrated at the six-leaf stage and at 9 d.p.i. and leaves at three different positions (top T, middle M, and bottom B) were collected. Fifty and twenty five micrograms of TSP extracted from leaves expressing Nef, Nef/AMCV-P19 or Nef/TBSV-P19 and Nef/PVX-P25 were loaded on the ELISA plate as four indipendent replicas. The amount of plant extract loaded on ELISA for each sample was calculated to be in the linear range of the standard curve. Nef expression was detected following the same ELISA procedure described above. Plant recombinant Nef expression levels, indicated as percentage of TSP, were estimated using a standard curve obtained from serial dilutions of bacterially-derived Nef (400 ng to 25 ng) (EVA#650, NIBSC-CFAR MRC).

### Statistical analysis

The data obtained were subjected to analysis of variance (ANOVA) using JMP8 Statistical Discovery Software (SAS Institute Inc, Cary, NC). Multiple comparisons among means were performed using each pair Student's t test.

### Nef siRNAs detection

Total RNA was extracted from frozen leaf tissues using mirVana miRNA isolation Kit (Ambion, Applied Biosystems, USA), according to the manufacturer's instructions. Briefly, frozen leaf samples were ground to a fine powder in liquid nitrogen and resuspended in 10 volumes of lysis/binding buffer plus 1/10 volume of miRNA homogenate additive. Organic extraction was performed with a volume of acid-phenol:chloroform (24:1) and RNA was isolated following the procedure for total RNA isolation. RNA concentration and quality were evaluated measuring the adsorbance at 260 nm (A_260_) and 280 nm (A_280_). For Northern blot analysis of siRNAs, 15 μg of total RNA were separated on denaturing 15% (w/v) polyacrylamide gel with 8 M urea, stained with ethidium bromide to display relative amounts of rRNA and transferred to positively charged nylon membrane (Roche, Germany). Si RNA Marker (New England Biolabs, USA), constituted by a set of three annealed synthetic siRNA duplexes that are 17, 21 and 25 base pairs in length, was used as low molecular weight RNA marker. Nef specific digoxigenin-labelled RNA (+) probe (618 nt) was transcribed from linearized plasmid using DIG RNA Labeling Kit (Roche Applied Science, Germany). Hybridization was performed overnight at 40°C, after hybridization the nylon membrane was washed in 2× SSC-0.2% SDS at 40°C. Probe detection was performed using the CDP-*Star *substrate (Roche, Germany) according to the manufacturer's protocol. The nylon membrane was exposed to hyperfilm ECL (GE Healthcare, UK) and developed with Kodak GBX developer/replenisher (Sigma-Aldrich, USA) for 2 min.

### Purification and Western-blot analysis of plant expressed Nef

Ten grams of transgenic *N. tabacum *(cv. SR1) or agroinfiltrated *N. benthamiana *leaves were ground in liquid nitrogen in a pre-cooled mortar and pestle. The samples were thawed in 3 ml of cold buffer A (50 mM sodium phosphate, 300 mM NaCl, 10 mM 2-betamercaptoethanol, pH 8.0 + protease inhibitors, Complete™ EDTA Free, Roche, Germany) per gram of plant material. The samples were centrifuged at 12000 g for 30 minutes at 4°C to pellet tissue debris. The clarified samples were mixed with 2 ml of Nickel resin (Qiagen, USA) equilibrated in buffer A following the manufacturer's instructions. Briefly, each sample was added to the resin and mixed at 4°C for 1 hour. The mixture was loaded onto a disposable plastic column (Bio-Rad, Hercules, CA). The column was washed twice with 10 bed volumes of buffer A and the sample eluted with 2.0 ml of elution buffer (buffer A + 10 mM imidazole). Four elution fractions of 0.5 ml were collected. Aliquots of eluted fraction were analysed by Western-blot to identify those containing Nef. Fractions were then loaded onto a PD-10 Desalting column (GE Healthcare, UK) to equilibrate the sample in buffer A. The second step of protein purification was performed using 2 ml of Talon™ cobalt resin (Clontech, USA) that specifically binds to His-tag, following the same procedure and buffers described for Nickel resin. Aliquots (20 μl) from each eluted fraction were separated on reducing SDS-12% (w/v) PAGE, and then electroblotted to PVDF membrane (Millipore, USA) using a Semi-Dry Transfer Unit (Hoefer TE70, GE Healthcare, Freiburg, Germany) at 40 mA, 50 V and 100 W for 1 h. Membranes were blocked overnight with 4% (w/v) dry milk in PBS before adding the mAb to HIV-1 Nef (EVA#3067.4, NIH AIDS Research and Reference Reagent Program) diluted 1:1000 in 2% (w/v) dry milk in PBS. The membrane was incubated for 2 h at room temperature. After three washes of 10 min with 0.1% (v/v) Tween 20 in and two washes of 5 min in PBS, the anti-mouse HRP-conjugated (NXA931 GE Healthcare, UK) was added at a dilution 1:2500 in 2% (w/v) dry milk in PBS. The membrane was incubated for 1 h at room temperature. Plant-derived Nef yields, indicated as ng/g of fresh tissue, were estimated by densitometric analysis of bands obtained in Western-blots. Plant-purified Nef as well as three different amounts (12.5, 25, 50 ng) of bacterially-derived Nef (EVA#650, NIBSC-CFAR MRC) were loaded on reducing SDS-12% (w/v) PAGE and analysed by Western-blot as described before. Band densities were determined from 16-bit grayscale TIFF format images, using the image J v.1.36b software (Image processing and analysis in Java, http://rsb.info.nih.gov/ij/) and Nef concentration was estimated using the standard curve obtained from the calculated band densities of the different dilutions of bacterially-derived Nef. The amount of plant purified Nef loaded on gel was calculated to be in the range of the standard curve.

### Mass spectrometry and protein identification

Bands from SDS-PAGE were manually excised, triturated, reduced, S-alkylated and digested over night at 37°C with modified trypsin (Sigma-Aldrich, USA). Gel particles were extracted with 25 mM NH_4_HCO_3_/ACN 1:1 and peptide mixtures were concentrated. Samples were desalted using Cleanup C18 Pipette Tips (Agilent Technologies, Santa Clara CA, USA), according to the manufacturer's instructions. Peptide mixtures were analysed by nLC-ESI-IT-MS/MS using HPLC-Chip/MS (Agilent Technologies, Santa Clara CA, USA), equipped with an Agilent HPLC 1200 series system with micro well-plate autosampler, capillary pump, HPLC-chip-cube interface and LC/MSD Trap XCT Ultra. The MS/MS scan range was between m/z 200 and 1800 for fragment ions. Spectra for peptides were subjected to protein identification as described before [30]. Briefly, database search was performed against NCBInr, by applying auto-validation criteria in the Spectrum Mill software (Rev A.03.02.06; Agilent Technologies, Santa Clara CA, USA). In order to minimize the number of false-positive hits, all MS/MS spectra were also searched against the reversed entries of the used databases. Only spectra with a reversed score at least two fold smaller than the real score were taken into account for the auto-validation. Moreover, the minimum difference between the scores of the top and second highest scoring database hit was at least 2-fold. After the auto-validation, only the subset of already identified proteins was used to search databases again, also allowing variable modifications, such as oxidized methionine.

## Authors' contributions

**RL **and **PC **cloned *amcv-p19 *and performed the agroinfiltrations and ELISA experiments; **RL **contributed to set up Nef purification protocol; **MEV **and **VC **performed Western-blot experiments; **GB **contributed to set up Nef purification protocol; **LN **performed statistical analysis; **LB **performed Mass spectrometry and protein identification; **EB **is the Group Coordinator; **MD **conceived the project and wrote the manuscript; **CM **performed the siRNA experiments, conceived the project and wrote the manuscript.

All authors read and approved the final manuscript.
